# Women’s experiences toward cervical cancer care at the initiation of care: a qualitative study closing the gap policy and implementation in Indonesia

**DOI:** 10.1186/s12905-026-04493-0

**Published:** 2026-05-13

**Authors:** Tuti Pahria, Hartiah Haroen, Hana Rizmadewi Agustina, Citra Windani Mambang Sari, Windy Natasya, Gatot Nyarumenteng Adhipurnawan Winarno, Sidik Maulana, Jerico Franciscus Pardosi

**Affiliations:** 1 Department of Medical-Surgical Nursing, Faculty of Nursing, Universitas Padjadjaran, Sumedang, Indonesia; 2 Department of Community Health Nursing, Faculty of Nursing, Universitas Padjadjaran, Sumedang, Indonesia; 3 Department of Fundamental Nursing, Faculty of Nursing, Universitas Padjadjaran, Sumedang, Indonesia; 4 Palliative Care Unit, Dr. Hasan Sadikin General Hospital, Bandung, Indonesia; 5 Department of Obstetrics and Gynecology, Faculty of Medicine, Universitas Padjadjaran, Dr. Hasan Sadikin General Hospital, Bandung, Indonesia; 6 Doctoral of Nursing Program, Faculty of Nursing, Universitas Padjadjaran, Sumedang, Indonesia; 7 School of Public Health and Social Work, Queensland University of Technology, Brisbane, Australia; 8 Faculty of Nursing, Universitas Padjadjaran, Jl. Ir. Soekarno KM. 21, Jatinangor, Sumedang, West Java 45463 Indonesia

**Keywords:** Cervical cancer; experience, Need, Early stage, Cancer care, SDGs-3

## Abstract

**Introduction:**

Cervical cancer remains a major public health concern in Indonesia despite ongoing national efforts toward elimination. While policy initiatives have expanded screening and treatment services, limited evidence exists regarding women’s lived experiences during the early stage of cervical cancer care. Understanding women’s perspectives is essential to strengthening a national care strategy.

**Objective:**

To explore women’s experiences during the early stage of cervical cancer care in Indonesia, from symptom recognition and abnormal screening results to diagnosis and initiation of treatment.

**Methods:**

A qualitative descriptive study was conducted in a national tertiary referral center in West Java, Indonesia. Eighteen women diagnosed with cervical cancer were recruited using purposive sampling to capture diverse clinical and experiential backgrounds. Data were collected through in-depth, semi-structured interviews between August and October 2025. Interviews were transcribed verbatim and analyzed using Braun and Clarke’s thematic analysis framework. Trustworthiness was ensured through member checking, audit trails, and collaborative theme validation.

**Results:**

Three themes emerged: (1) women’s experiences in accessing care services, (2) difficulties and coping mechanisms, and (3) unmet informational and psychosocial needs. Participants described normalization of early symptoms, misinterpretation at primary care level, fragmented referrals, and inconsistent staging information, contributing to diagnostic uncertainty and emotional distress. Although national health financing covered treatment costs, indirect financial burdens persisted. Women actively sought simplified explanations through digital platforms to compensate for communication gaps. Many entered treatment with limited preparation and inadequate counseling regarding side effects.

**Conclusion:**

Initiation of care represents a critical bottleneck within Indonesia’s cervical cancer elimination efforts. Strengthening early clinical recognition by healthcare providers, referral continuity, structured counseling, early palliative care integration, and digital navigation systems is essential to bridge the policy-implementation gap and support achievement of the WHO cervical cancer elimination target of treating 90% of women diagnosed with cervical disease.

**Supplementary Information:**

The online version contains supplementary material available at 10.1186/s12905-026-04493-0.

## Introduction

Cervical cancer remains one of the ten leading causes of mortality among women of reproductive and productive age worldwide [1]. Cervical cancer is most commonly caused by one of about 15 genotypes of carcinogenic persistent infection of human papillomavirus (HPV), particularly HPV-16 and HPV-18 [2, 3]. Despite being largely preventable, the global burden of the disease continues to rise, with deaths projected to increase by nearly 70% in the coming decades if current trends persist [1]. This increase is disproportionately concentrated in low- and middle-income countries (LMICs), where the majority of new cases and deaths occur [1, 4]. Although effective preventive strategies exist, including HPV vaccination, regular screening, and early treatment of precancerous lesions, significant challenges remain. Persistent inequities in access to vaccination, screening services, diagnostic facilities, timely treatment, and palliative care continue to widen the survival gap between high-income and resource-limited settings [5].

The growing global burden of cervical cancer has prompted an urgent international call to action. In response, the World Health Organization (WHO) launched *the Global Strategy to Accelerate the Elimination of Cervical Cancer* as a Public Health Problem, setting the ambitious 90–70–90 targets to be achieved by 2030: 90% of girls fully vaccinated with the HPV vaccine by age 15, 70% of women screened with a high-performance test at least twice in their lifetime, and 90% of women identified with cervical disease receiving appropriate treatment and care [6]. In Indonesia, efforts to advance toward the elimination of cervical cancer have been gathering momentum [7]. The government has formalized the National Cervical Cancer Elimination Plan 2023–2030, strengthening multi-sector partnerships and scaling up HPV vaccination, screening, and treatment services nationally [8]. Despite these advancements, substantial gaps remain, especially in treatment access and service delivery across the archipelago [9, 10]. Access to timely and comprehensive treatment for cervical disease also varies widely by geography and facility capacity, with care and treatment services unevenly available outside urban centers [11–13].

Addressing persistent gaps in cervical cancer treatment requires strong national commitment to develop a comprehensive and women-centered national care strategy. National strategies have largely emphasized coverage indicators [14]; however, less attention has been given to women’s lived experiences as they navigate the health system after receiving abnormal screening results or a confirmed diagnosis. Understanding these early care trajectories is critical, as delays may compromise treatment outcomes, such as effects to prolong the interval between external beam radiation therapy and brachytherapy [15]. A study by Tjokroprawiro et al. quantitatively reported that most women presented at advanced stages, with low awareness of prevention, limited screening, and frequent delays due to complex referral pathways, contributing to delayed diagnosis and treatment initiation [16]. While this study provided important insights into screening program challenges, it primarily examined service delivery from a programmatic perspective rather than women’s lived experiences during early diagnostic and treatment pathways.

Therefore, this study aims to explore women’s experiences and perceived needs during the early stage of cervical cancer care in Indonesia, particularly from the point of abnormal screening results or symptom recognition to diagnosis and initiation of treatment. The findings are expected to provide a new women-informed perspective to strengthen Indonesia’s national cervical cancer care framework.

## Methods

### Study design

This study was a qualitative descriptive design to explore the experiences of women with cervical cancer at the initiation of care. Initiation of care in this study is defined as the period from symptom recognition or abnormal screening results to confirmed diagnosis (cervical pre-cancer and cervical cancer) and the start of first-line treatment, such as cryotherapy, thermal ablation, conization, surgical procedure, radiotherapy, and chemotherapy [8, 17]. The study reporting followed the Standards for Reporting Qualitative Research (SRQR) guidelines [18], the checklist of reporting provided in the supplementary File 1.

### Study setting

This study was conducted at Dr. Hasan Sadikin General Hospital, Indonesia, a major tertiary referral and central cancer treatment hospital serving patients from West Java and surrounding regions. The hospital functions as a national referral center for oncology care, including comprehensive cervical cancer management such as diagnosis, surgery, chemotherapy, radiotherapy, and follow-up services. Participants were recruited from the oncology outpatient department at the hospital. Data collection was carried out between August and October 2025.

### Participants and sampling

Eighteen women diagnosed with cervical cancer were recruited using purposive sampling to capture diverse clinical and experiential backgrounds across the cervical cancer care pathway. Participants included women who were currently undergoing treatment as well as those who had completed one or more phases of care, such as diagnosis, surgery, chemotherapy, radiotherapy, and follow-up. Women were identified through the oncology outpatient department of the hospital. Eligible participants were approached directly during their routine clinical visits or through coordination with healthcare staff who assisted in introducing the study to potential participants. Written informed consent was obtained from all participants prior to data collection.

Enrollment continued until thematic saturation was achieved to ensure sufficient depth and variability of data. Saturation was considered reached when additional interviews yielded no substantially new insights. Two additional interviews were conducted to confirm consistency and strengthen the richness of participants’ experiences across themes.

### Inclusion and exclusion criteria

Participants were eligible for inclusion if they met the following criteria: (1) women diagnosed with cervical cancer who were either currently undergoing treatment or had completed at least one phase of care (diagnosis, therapy, or follow-up); (2) aged 18 years or older; (3) able to communicate clearly in Bahasa Indonesia; and (4) willing to participate voluntarily and provide written informed consent.

Women were excluded if they were (1) in a critical clinical condition that prevented participation in an interview; (2) had cognitive or communication impairments limiting their ability to provide informed responses; or (3) declined or withdrew consent at any stage of the study.

### Data collection

In-depth interviews were conducted face-to-face in Bahasa Indonesia at locations agreed upon by the participants, primarily within the Oncology Outpatient Department at Dr. Hasan Sadikin General Hospital, Bandung. The setting was arranged to ensure privacy, comfort, and emotional safety during the discussions. Interviews were conducted by the two authors (TP and HH), senior lecturers and researchers who have prior experience in qualitative health research, and were supported by trained research assistants to facilitate note-taking and logistical coordination. A semi-structured interview guide informed by concepts of patient care pathways and health-seeking behavior in cancer care (Supplementary File 2). Each interview lasted approximately 40–60 min and was audio-recorded with participant consent. Field notes were taken to capture contextual details and nonverbal expressions that complemented the verbal data.

All interviews were conducted in Bahasa Indonesia, the participants’ native language. Audio recordings were transcribed verbatim and subsequently translated into English by the first author and verified by a bilingual researcher (HRA) to ensure linguistic accuracy and preservation of contextual meaning. All transcripts and translated documents were stored securely in password-protected folders accessible only to the research team. The first author’s background in health and women-centered research facilitated rapport-building with participants; however, reflexive journaling was maintained throughout the data collection process to minimize potential bias and ensure that interpretations remained grounded in participants’ narratives.

### Data analysis

The data were analyzed using thematic analysis following the six-step approach proposed by Braun and Clarke [19]. Thematic analysis enabled a flexible yet rigorous interpretation of participants’ narratives across different stages of the cervical cancer care pathway. The analysis proceeded through six stages: (1) all interview recordings were transcribed verbatim to ensure a complete representation of participants’ accounts; (2) transcripts were read repeatedly to achieve data familiarization and immersion; (3) initial codes were generated by identifying meaningful segments related to experiences, challenges, information needs, emotional responses, and expectations toward digital support; (4) codes were organized into potential themes and sub-themes reflecting shared patterns across participants; (5) themes were reviewed, refined, and clearly defined to ensure internal consistency and conceptual clarity; and (6) the final themes were interpreted and mapped to identify practical implications for the design of a digital navigation system, including informational, psychosocial, clinical, and service-navigation features. The coding and analysis were conducted manually to maintain close engagement with the data and ensure nuanced interpretation. To enhance credibility, the research team discussed and validated the themes collaboratively. The final thematic framework served not only to describe women’s experiences but also to translate these findings into functional directions for designing a digital navigation system tailored to cervical cancer care in Indonesia.

### Trustworthiness

Trustworthiness in this study was ensured by applying Guba and Lincoln’s criteria: credibility, transferability, dependability, and confirmability [20]. Credibility was established through prolonged engagement with participants across treatment and shelter settings, allowing the researchers to gain a comprehensive understanding of their lived experiences. Member checking was conducted during the interview by periodically summarizing participants’ responses and again at the end of each session to confirm key points and interpretations, ensuring accuracy and authenticity of the data. Transferability was supported by providing detailed descriptions of the study setting, participant characteristics, data collection procedures, and analytical processes, enabling readers to assess the applicability of the findings to similar contexts. Dependability was addressed by maintaining a systematic audit trail documenting all methodological decisions, coding steps, and theme development throughout the study. Confirmability was strengthened by preserving raw interview transcripts, field notes, and coding records, as well as conducting collaborative discussions among the research team to ensure that interpretations remained grounded in participants’ narratives rather than researcher assumptions.

### Ethical considerations

Ethical approval for this study was granted by the Research Ethics Committee. This study was conducted in accordance with the principles of the Declaration of Helsinki. All participants provided written informed consent after being fully informed about the study’s objectives and their rights. Confidentiality was maintained through the use of pseudonyms, and data were securely stored on password-protected devices accessible only to the research team.

## Results

### Characteristics of participants

A total of 18 women with cervical cancer participated in this study (Table 1). The mean age of participants was 35.4 years (SD = 8.35), indicating that most were in their reproductive years. In terms of educational background, the majority had completed junior high school (55.6%). All participants were unemployed (100%). Most were married (88.9%), and the dominant living arrangement was with their spouse (77.8%), while a smaller proportion lived with children (16.7%) or extended family (5.6%). Regarding clinical characteristics, most participants were diagnosed at an early stage of cervical cancer (72.2%), whereas 27.8% were in an advanced stage. Nearly all participants had experienced hospitalization (94.4%). All participants were covered under public health insurance (*the Jaminan Kesehatan Nasional*/ JKN) (100%).

**Table 1:** Characteristics of participants

Characteristic	Frequency (*n*)	Percentage (%)
Age (Mean, SD)	35.4 (8.35)	
Education		
Elementary school	2	11.1
Junior high school	10	55.6
Senior high school	4	22.2
College	2	11.1
Employment status		
Unemployed	18	100
Marital status		
Married	16	88.9
Single/widow	2	11.1
Living with		
Spouse, grandchild	1	5.6
Child	3	16.7
Spouse	14	77.8
Cancer stage		
Early	13	72.2
Advanced	5	27.8
Ever hospitalized		
Yes	17	94.4
No	1	5.6
Health insurance coverage		
Public (JKN)	18	100

*JKN * Jaminan Kesehatan Nasional*,SD * standard deviation

### Qualitative findings

A total of three themes emerged from participants’ narratives regarding their lived experiences throughout cervical cancer at the early stage of treatment (see Table 2).

**Table 2:** Themes, Sub-themes, and quotations of participants

Themes	Sub-themes	Quotation source
Women’s experience in accessing care services	Lack of awareness	P5, P9
	Lack of information	P1, P3
	Uncertainty of disease diagnosis	P11, P12, P13
	Information seeking through the internet	P9, P10
	Utilize the benefit of universal health coverage	P5
Women’s difficulties-experiences and their coping mechanisms	Financial difficulties	P2, P8
	Coping mechanisms towards cancer-related symptoms	P1, P7, P15
	Emotional experience	P17, P18
	Spiritual and social support	P13, P18
Women’s needs	Information Need	P3, P7
	Social support needs	P2, P18

### Theme 1: women’s experience in accessing care services

#### Lack of awareness

Participants commonly described experiencing abnormal vaginal discharge and persistent bleeding long before seeking medical attention. These symptoms were often perceived as normal reproductive changes, hormonal imbalance, or menopause-related conditions. As a result, many delayed consulting healthcare providers until the symptoms worsened, became prolonged, or interfered with daily functioning. This normalization of early warning signs contributed to late recognition and postponed diagnosis.

“At first, the discharge was watery… then the abnormal bleeding didn’t stop… I thought it was hormonal… until the biopsy showed stage 2B.” (P5).

“I thought it was menopause… bleeding for two weeks until I fainted… only then I found out it was stage 3B.” (P9).

#### Lack of information

Participants demonstrated very limited baseline knowledge about cervical cancer. Participants expressed confusion about how the disease develops and admitted they had never previously received information about cervical cancer symptoms or risk factors. This lack of awareness contributed to delayed care-seeking and difficulty comprehending the seriousness of their condition even after diagnosis

“I don’t know… I’m shocked… I don’t know the causes or symptoms.” (P1).

“I don’t know how cervical cancer starts.” (P3).

#### Uncertainty of disease diagnosis

The pathway to diagnosis was often long, fragmented, and marked by misinterpretation by healthcare providers. Participants reported repeated visits to different doctors or health centers where their symptoms were initially diagnosed as urinary tract infections or inflammation. The prolonged trial-and-error process, including delayed biopsy and referral, intensified uncertainty and frustration. Participants also highlighted inconsistencies in staging between hospitals, which further complicated their understanding and emotional response.

“Two doctors said it was a urinary tract infection… only the third doctor found a 6 cm lump.” (P11).

“They gave antibiotics for three months before asking for a biopsy.” (P12).

“At X hospital it was stage 2, but at Y hospital it became 3B.” (P13).

#### Information seeking through the internet

Participants independently sought health information through digital platforms, such as ChatGPT, Google search, and Youtube, as these source provided simplified and summarized explanations.

“Searching via ChatGPT… it summarizes everything.” (P9).

“I often look on YouTube and Google about herbal treatments; they say it has been tested in Korea…. I understand more from YouTube; the doctors there explain things very clearly.” (P10).

#### Utilize the benefit of universal health coverage

Despite these barriers, financial coverage by JKN and strong family encouragement motivated participants to continue treatment.

“JKN helps, no financial problems.” (P5).

### Themes 2: women’s difficulties-experiences and their coping mechanisms

#### Financial difficulties

Participants described structural challenges such as repeated hospital visits, transportation difficulties, and high non-medical costs. Although JKN covered treatment expenses, participants still struggled with indirect costs such as accommodation and daily living needs.

“Distance and daily costs are burdensome.” (P8)

“JKN covers medical costs, but daily expenses are difficult.” (P2).

#### Coping mechanisms towards cancer-related symptoms

To cope with cancer-related symptoms, participants adopted various self-care strategies, including dietary adjustments as well as psychological and spiritual coping, such as reciting “*istighfar*” to manage anxiety.

“I still eat small amounts even though I’m nauseous.” (P7).

“When I feel nauseous, I drink ginger.” (P15)

“I do feel anxious… I cope with it by reciting “istighfar” (a form of Islamic prayer seeking forgiveness from God)” (P1).

#### Emotional experience

Diagnosis triggered profound psychological responses including shock, fear, stress, and resignation. Many participants coped by surrendering to religious beliefs and accepting their illness as destiny. Emotional vulnerability was particularly evident during the early phase after diagnosis.

“I was shocked… stressed… resigned… it’s God’s will.” (P18).

“I just surrender, the important thing is treatment.” (P17).

#### Spiritual and social support

Faith and community support, especially from shelter companions, played an essential role in strengthening participants’ resilience during treatment.

“Keep praying… may you be blessed with health.” (P18).

“At the shelter, companions help with cooking.” (P13).

### Theme 3: women’s needs

#### Information Need

Participants strongly emphasized the need for clear, structured, and trustworthy information regarding their diagnosis. They wanted comprehensive explanations about symptoms, disease progression, and available medical interventions. Participants also expressed doubts about information delivered by junior doctors and desired confirmation from senior physicians to increase confidence and reduce uncertainty.

“I want to know everything… symptoms, causes, treatment.” (P3).

“I want confirmation from a senior doctor, not only residents.” (P7).

Participants described entering treatment with minimal preparation and insufficient counseling. Many felt shocked by the severity of radiation and chemotherapy side effects, which they had not anticipated. This lack of information heightened anxiety and physical distress during therapy.

“I didn’t know the side effects would be like this… I feel nauseous all the time.” (P14).

“I need information about radiation… chemo… everything.” (P4).

#### Social support need

Participants emphasized the importance of emotional reassurance from family and healthcare providers. They expressed a strong desire to be listened to empathetically rather than treated merely as clinical cases.

“I want to be heard… not just asked questions.” (P2).

“…My children tell me to stay strong.” (P18).

## Discussion

This study explored women’s experiences at the initiation of cervical cancer care in Indonesia. The findings of this study provide three key contributions to understanding cervical cancer care initiation in Indonesia. First, it highlights persistent gaps at the facility level, including low sensitivity of healthcare providers that contribute to late recognition and diagnostic uncertainty. Second, it reveals fragmentation within the diagnostic and referral process, characterized by repeated consultations, inconsistent staging information, and limited communication, which may weaken timely linkage to care despite universal health insurance coverage. Third, it identifies substantial unmet informational and psychosocial needs at the point of diagnosis, alongside emerging women-driven digital information-seeking behaviors.

Previous research by Tjokroprawiro et al. [16] reported late-stage presentation, low awareness of prevention, minimal prior screening, prolonged delays exceeding 12 months, and multiple referral visits before women reached tertiary care. While those findings clearly describe the magnitude of delay and structural barriers within the system, the present study extends this evidence by providing insight into the lived experiences that shape those trajectories at the initiation of care. The findings demonstrate how early symptoms were normalized, how repeated misinterpretation by healthcare providers contributed to diagnostic uncertainty, and how inconsistent staging information intensified emotional distress and confusion. Additionally, this study reveals substantial informational and psychosocial needs at the point of diagnosis, as well as emerging women’s reliance on digital platforms to compensate for communication gaps. ​​The findings of this study can be interpreted within the framework of Indonesia’s National Cervical Cancer Elimination Plan 2023–2030 [8]. The framework is structured around four pillars: service delivery; education, training, and outreach; enablers of progress; and stewardship and coordination [8]. The findings of this study suggest that gaps at the initiation of care intersect with each of these pillars.

Most of the women in this present study reported that they presented with postmenopausal bleeding and abnormal vaginal discharge, similar to other studies [21, 22]. In Indonesia, cervical cancer screening is largely delivered through opportunistic approaches within referral healthcare settings, without a nationwide population-based call-recall system, which may contribute to suboptimal coverage and delayed detection. Additionally, in this present study, participants described receiving antibiotics for months before biopsy confirmation, being referred across multiple facilities, and experiencing confusion due to differing explanations between hospitals. Although tertiary oncology services were ultimately accessible, the pathway toward confirmed diagnosis and treatment initiation was frequently emotionally distressing. These kinds of healthcare provider-related delays have largely been examined in relation to providers’ training and their familiarity with cervical cancer screening guidelines and policy frameworks [23, 24]. The documented delays and diagnostic inconsistencies highlight the importance of Strategy 3.2, which calls for strengthening pathology services to ensure timely and accurate diagnosis [8]. Fragmented referral pathways directly relate to Strategy 3.1, emphasizing the need to strengthen overall treatment service capacity and coordination across levels of care. These patterns illustrate that the trajectory towards the national cervical cancer elimination needs continued attention.

In Indonesia, the JKN is part of universal health coverage reform and national health financing. It offers extensive benefits that cover advanced health services, such as cancer therapies [25]. However, the findings further highlight the relevance of the enablers of progress pillar. Although all participants were covered under JKN, indirect costs such as transportation, accommodation, and daily living expenses remained substantial burdens. This indicates that health financing alone may not be sufficient to ensure equitable access to timely care [26, 27]. In addition, women’s reliance on digital platforms to obtain information suggests an opportunity to strengthen system-level enablers through structured digital navigation tools. Leveraging digital health platforms may help bridge communication gaps, provide reliable information, and support coordinated linkage to care, particularly during the emotionally vulnerable initiation phase [28, 29]. The stewardship and coordination pillar becomes particularly relevant in addressing the fragmented transitions described by participants [8]. Strengthening governance mechanisms, standardizing referral pathways, and integrating digital navigation systems may enhance continuity of care and ensure that policy commitments translate into consistent implementation across regions [30, 31].

Finally, the findings suggest that the initiation of care represents a critical bottleneck within cervical cancer elimination efforts in Indonesia. Although national policies have expanded screening and treatment capacity [8, 32], the transitional phase between symptom recognition, diagnosis, and treatment initiation remains inadequate. Integrating structured patient navigation and earlier supportive care, including elements of early palliative care such as symptom management and psychological support, may help address these gaps [33–35]. Early palliative approaches should not be viewed solely as end-of-life care but as comprehensive supportive care initiated at diagnosis to improve treatment readiness, coping, and quality of life [36].

The findings identify care initiation as a critical operational gap where timely diagnosis, referral coordination, and treatment readiness frequently falter. To strengthen alignment with the 90% treatment objective, several priority actions are required: (1) Strengthening early clinical recognition by healthcare providers, while strengthening pathology turnaround time and inter-facility communication to ensure timely treatment decisions; (2) institutionalizing structured counseling and treatment preparation protocols at the point of diagnosis; (3) integrating early supportive or palliative care components to address symptom burden and psychological distress; and (4) implementing digital navigation systems to facilitate real-time referral tracking, patient education, and continuity of care across levels of service.

### Limitation

These findings should be interpreted considering the following limitations. First, participants were recruited from a single tertiary referral hospital, which may limit the transferability of findings to women receiving care in rural or lower-level facilities. Women who successfully reached tertiary services may differ from those who never accessed referral care, potentially underrepresenting more severe barriers to linkage. Second, most participants were already engaged in treatment at the time of interview, which may have influenced retrospective recall of early experiences. Third, although efforts were made to ensure trustworthiness through member checking and team-based analysis, the qualitative design inherently reflects subjective interpretations shaped by participants’ narratives and researcher reflxivity. Fourth, the translation of the interview may be biased due to the fact that it was undertaken by the researcher. Fifth, this study did not specifically explore the experiences of women living with HIV, who may face distinct clinical, social, and structural barriers in cervical cancer care [37]. Given the higher risk of cervical cancer progression among women with HIV and the need for integrated HIV-cervical cancer services, barriers and strategies for care initiation may differ substantially in this population [38]. Finally, the study focused on women perspectives and did not include healthcare provider or policymaker views, which may provide complementary insight into system-level constraints.

## Conclusion

The initiation of cervical cancer care represents a critical gap within the national elimination strategy in Indonesia. Despite expanded screening and treatment capacity, women continue to experience fragmented referrals, delayed diagnostic confirmation, limited communication, and unmet psychosocial needs at the point of care transition. Indonesia needs to strengthen implementations of the treatment pillar by ​​strengthening early clinical recognition by healthcare providers, enhancing structured patient counseling, and integrating digital navigation and early supportive care mechanisms. Addressing these operational gaps is essential to ensure that national policy commitments effectively translate into achieving the 90% treatment target and advancing equitable cervical cancer elimination.

## Supplementary Information

Supplementary Material 1.

Supplementary Material 2.

## Data Availability

The data that support the findings of this study are available on request from the first author, TP.
