# Supplementary material for: Women’s experiences toward cervical cancer care at the initiation of care: a qualitative study closing the gap policy and implementation in Indonesia
Source: BMC Womens Health. 2026 May 13;26:339. doi: 10.1186/s12905-026-04493-0 (PMC13343666; doi:10.1186/s12905-026-04493-0)
Supplement: Supplementary file 2 — Supplementary Material 2. [file 12905_2026_4493_MOESM2_ESM.pdf]

Interview Guideline

Topic: Women's Experiences in Seeking and Starting Cervical Cancer Care

Opening

Thank you for agreeing to participate. We would like to understand your experience from the time you first noticed something about your health until you started treatment. Please feel free to share your story in your own words. There are no right or wrong answers.

Main Narrative Question

Can you tell me the story of what happened from the beginning until you started treatment?

(*Allow uninterrupted narration first*.)

Flexible Probing Questions

(*Use only if needed to deepen understanding, not to lead.*)

A. Early Experience
   - Can you tell me more about what was happening at that time?
   - What were your thoughts when that happened?
   - What did you do next?

B. Healthcare Journey
   - What happened when you first visited a health facility?
   - And after that?
   - How was your experience during those visits?
   - Were there any moments that were particularly important to you?

C. Personal Impact
   - How did this experience affect you?
   - What was most challenging during this period?
   - What helped you continue?

D. Support and Resources
   - Who or what supported you during this time?

- Did you seek help or advice from anyone?
- Is there anything that made the process easier or harder?

E. Reflection
   - Looking back, how do you see this whole experience?
   - Is there anything that could have been improved?
   - What would you suggest for other women in similar situations?

Closing

Is there anything else you would like to share? Thank you very much for sharing your experience.
